# Salicylate Poisoning Potential of Topical Pain Relief Agents: From Age Old Remedies to Engineered Smart Patches

**DOI:** 10.3390/medicines4030048

**Published:** 2017-06-30

**Authors:** Ashleigh Anderson, Aaron McConville, Laura Fanthorpe, James Davis

**Affiliations:** School of Engineering, Ulster University, Jordanstown, Northern Ireland BT37 0QB, UK; Anderson-A2@ulster.ac.uk (A.A.); Mcconville-a4@ulster.ac.uk (A.M.); Fanthorpe-l@ulster.ac.uk (L.F.)

**Keywords:** smart patches, methyl salicylate, toxicity, microneedle, transdermal

## Abstract

The pain relief capabilities of methyl salicylate are well established and a multitude of over-the-counter products populate pharmacy shelves. Over-application of the topical preparation containing the drug, or its accidental ingestion, invariably result in salicylate poisoning and in severe cases can be fatal. The drug has been a regular feature of the US National Poison Database Survey over the past decade and continues to pose a risk to children and adults alike. The aim of the review has been to cast a spotlight on the drug and assess why its use remains problematic, how technology could offer more efficacious delivery regimes, and minimise the possibility of accidental or intentional misuse.

## 1. Introduction

It has become common practice for patients suffering from musculoskeletal injuries or disease to seek non-prescription medicines in an attempt to minimise pain and ease their condition [1,2,3]. Recent estimates suggest that those suffering from acute or chronic pain in the US number in the hundreds of millions [3] but, while some 10% of all Americans report suffering from chronic pain, the incidence increases to 60% when considering those aged 65 years or older [4]. Chronic pain can have a pernicious toll on quality of life and will affect both everyday family activities and workplace responsibilities, and it is one of the main causes of physical disability in the US [5]. Although the true impact may be impossible to quantify, some estimates place the annual socio-economic burden experienced by US citizens alone in the range of $600 billion [6]. Conventional treatment options for those with chronic musculoskeletal pain are nonsteroidal anti-inflammatory drugs (NSAIDS), opioids, or surgery which, in many cases, often fail to provide long-term benefit [7,8,9]. As a result, many will seek alternative treatments and it is little surprise to find, therefore, that there is a burgeoning market (valued at over $100 billion per annum (p.a.)) in the supply of non-prescription over-the-counter (OTC) products that purport to provide a wealth of therapeutic benefits [2]. 

**Figure 1:**
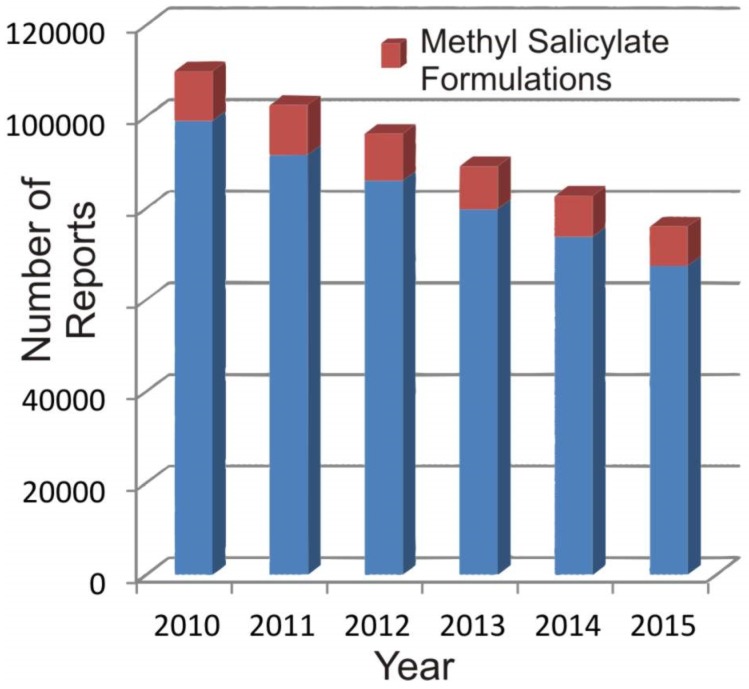
Annual case reports of suspected/potential poisoning due to the use of topical agents. Data extracted from the American Association of Poison Control Centres’ National Poison Data System [13,14,15,16,17,18].

The latter typically result from the formulation of either single entity or drug combinations that can impart a local analgesic, anaesthetic, antipruritic, or counterirritant action [10]. The ingredients can be derived from natural sources or through industrial synthesis and are incorporated into a diverse range of forms such as pills, gels, ointments, lotions, sprays and, more recently, dressings and transdermal patches [10,11]. The fact that these products can be readily acquired from sources that offer no qualified advice beyond the packaging instruction can, however, create issues over their efficacious application [12]. As such, the potential for misuse, accidental or deliberate, can be significant and is evidenced in the annual reports arising from the US National Poison Data System (NPDS) [13,14,15,16,17,18]. Salicylates feature regularly in the latter, as indicated in Figure 1, and although their therapeutic properties have long been recognised, the delivery methods have changed little since their discovery. The aim of the present communication is to explore new developments in the smart administration of topical agents. The principal focus is on methyl salicylate, given its prominence in the NPDS database, and although the specifics of the technological options are discussed relative to it, they are often generic and applicable across a spectrum of therapeutic agents. 

## 2. Historical Perspective

Methyl salicylate (MS) is a common, yet complex, signalling molecule used by a host of plant and tree species which can warn neighbours of herbivorous insect infestation thereby enabling the biochemical upregulation of defences and the recruitment of the insect’s predators [19]. A more common association within the wider public arena or among the wider public, however, relates to its use as an essential oil, fragrance, and medicinal compound [20]. Historically, it was extracted from the small wintergreen plant (*Gaultheria procumbens* L.), from which it gets its common name, and from birch trees (*Betula lenta* L.) [21]. The leaves and bark from the latter were used by indigenous peoples across America and Canada as the basis of herbal infusions for the treatment of rheumatism, fever, and gastrointestinal ailments not to mention as a topical agent for burns, wounds, and bruises [22]. The analysis of birch leaves reveal that they contain between 0.23–0.6% *w*/*w* of essential oil of which 99.8% is MS and, hence, provides some evidential weight to the professed analgesic properties of folklore remedies [22,23]. 

## 3. Commercial Application

The action of MS is multimodal with analgesic, anti-inflammatory and rubefacient/counterirritant properties. The former arises from the rapid hydrolysis of the ester yielding salicylic acid as the active agent [24,25,26] and, as such, its analgesic action is beyond question. It has a vasodilatory action upon absorption resulting in an increased localised blood flow and, consequently, produces a rise in tissue temperature—its rubefacient action [27]. Menthol, in contrast, has a cooling effect [28] and, thus, their dual incorporation can set up a counterirritant action [29]. The therapeutic efficacy of the latter is, however, more contentious, with the UK National Institutes for Clinical Excellence (NICE) reporting that there is no clinical evidence to support its use as such [30]. Similarly, the US FDA, with the exception of Salonpas patches®, has noted that there exists inadequate data to enable the recognition of effectiveness of such products for the specified OTC uses [30,31]. Nevertheless, MS has made the journey from herbal recipe to mainstream pharmaceutical, with synthetic manufacturing processes accounting for almost all modern-day formulations. While there are a multitude of pseudo-medicinal products in which MS is listed as the active agent, it must be recognised that it is also employed as a flavour and fragrance enhancer. The compound gives a sweet, mint-like odour and is frequently incorporated into breath mints and chewing gum and, given that it possesses some intrinsic antiseptic properties [32], has found use in toothpastes and mouthwashes. However, its use is much more pervasive with the “fresh” fragrance being employed in a range of common household disinfectants. The breadth of cosmetic products in which MS features is highlighted in Table 1, along with some of the typical concentrations disclosed to the US Food and Drug Administration database.

The concentrations of MS in standard cosmetic products tends to be very low, but can increase dramatically when considering preparations designated for therapeutic use. Aromatherapy is a prime example where oil of wintergreen is essentially 98% MS [22,23,33]. On the assumption that the practitioner is appropriately skilled and following their working codex, the wintergreen oils will normally be blended with other “base” oils such that the overall concentration falls within 0.5–5% [34,35]. Massage OTC products (typically in the form of liniments, creams, and dressings) utilise the anti-inflammatory properties of MS and, where once the preserve of those engaged in sport, are increasingly being marketed to those suffering from joint and muscle pains originating from rheumatic conditions [36]. These products, designed for topical application, typically have MS concentrations in the region of 3–20% and can come with minimal instruction or advice. Any drug product containing salicylates intended for oral ingestion must be appropriately labelled with a warning that misdirected use may be dangerous and that it must be kept out of the reach of children. The same only applies to topical MS products where the concentration of the latter exceeds 5% [37].

## 4. Transdermal Biochemistry

Oral NSAIDs are commonly used to treat musculoskeletal pain, but repeated administration in chronic conditions can give rise to a number of adverse effect profiles [38]. Topical administration has garnered considerable interest in recent years as the delivery mechanism provides a clear opportunity to avoid the complications of oral intake and, in particular, gastric irritation. The key requirement, however, is that the NSAID can sufficiently penetrate the skin and, therein, reach the affected site. There can be a degree of ambiguity over the mode of action relating to whether the clinical outcome arises from the direct translation of the drug locally to the affected tissue or whether it is the result of systemic absorption and subsequent redistribution [39]. The latter can only occur where there the drug can pass across the top layers of the skins (stratum corneum, viable epidermis, and basement layer) to reach the dermis which contains the blood vessels necessary for transport into the deeper tissues [24,40]. 

**Figure 2:**
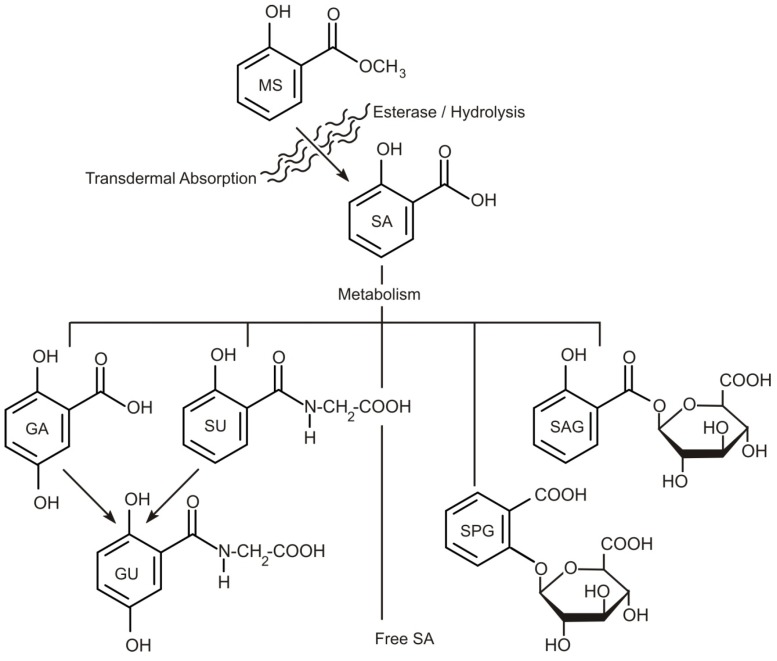
Metabolites of salicylic acid (SA); SPG: salicylic acid phenolic glucuronide; SAG: salicylic acid acyl glucuronide; SU: salicyluric acid; GA: gentisic acid; GU: Gentisuric acid [49,50].

Methyl salicylate is lipophilic and when applied as a topical agent has been shown to readily penetrate the skin and is readily hydrolysed to salicylic acid in the tissues [41,42,43,44,45,46,47,48]. Yano and colleagues (1991), however, demonstrated that menthol and camphor, added as co-drugs, significantly inhibit the esterase activity in a dose-dependent manner and, consequently, many of the topical massage products utilise these agents in their formulation [43]. Once absorbed, the resulting salicylate is distributed throughout the tissues and transcellular fluids, primarily through passive pH-dependent processes. It has been estimated that the plasma half-life for salicylate is 2 to 3 h in low doses, increasing to 12 h at usual anti-inflammatory doses. In cases where supratherapeutic doses/salicylate intoxication occurs, the half-life may be as much as 15 to 20 h [48]. Under normal therapeutic regimes, conjugation with glycine to form salicyluric acid and with glucuronic acid to form salicyl acyl and phenolic glucuronide are the major metabolic/excretion pathways as outlined in Figure 2. Oxidation of the salicylate to gentisic acid (2,5-dihydroxybenzoic acid), 2,3-dihydroxybenzoic and 2,3,5-trihydroxybenzoic acids can also occur, but are minor in comparison to the other routes [48]. The metabolites are readily excreted in the urine with the free unmodified salicylate accounting for 10–30%. The latter can rise significantly at large therapeutic loadings as both the glycine and glucuronide pathways have limited capacity and saturate easily [48]. As a result, the tissues become saturated and chronic salicylate toxicity can occur—hence, the prolonged half-life.

One of the more recent studies examining the transdermal influence of MS application in human volunteers was performed by Morra and colleagues (1996) [42]. An ointment containing 12.5% MS was applied to twelve volunteers (six male, six female) twice daily over a period of four days and the salicylate concentrations within serum analysed prior to dosing and at various intervals between subsequent applications. Urine was also collected during the entire study. While salicylate was found within the serum, at no point was unchanged MS detected, despite its relatively high loading within the ointment—confirming its rapid hydrolysis upon absorption. Serum salicylate concentrations ranged from 0.3–0.9 mg/L within the first hour of application and increased to 2–6 mg/L by day 4. Salicylic acid, along with its uric acid (SU) conjugates, were detected in the urine at concentrations of 15.6 and 491.9 mg/L, respectively. The relative proportions of the various metabolites in urine can be highly variable and dependent on both therapeutic dose and pH. The typical compositional breakdown under moderate doses yields: free salicylic acid (10%), salicyluric acid (75%), salicylic phenolic glucuronide (10%), salicylic acyl glucuronides 5%), and gentisic acid (less than 1%). Gilman (1990), however, demonstrated that under acid or alkaline conditions—the recovery of free salicylic acid could drift between 2 and 30% of the ingested drug [47]. The recovery of total salicylic acid over days 1–4 was 15.5, 22.0, 22.4, and 22.2%, respectively [42]. In humans, it has been estimated that 12–20% of MS applied topically is directly absorbed within the first 10 h, but it is important to note that the composition of the actual product (ethanol, isopropanol, menthol, camphor, etc.) along with the condition of the skin can greatly influence the transport and hydrolysis kinetics [42].

## 5. Accidental/Intentional Misuse

The ubiquity of salicylates in over-the-counter (OTC) topical pain medications has long given rise to concerns over their potential for misuse and accidental poisoning [51,52]. The high morbidity and mortality of paediatric referrals after ingestion of preparations containing oil of wintergreen has been of particular importance as, in its freshly distilled form, it contains over 98% MS [52,53,54,55,56]. It is widely established that the ingestion of a single teaspoon (~5 mL) of the oil, whether synthetic or natural, can be equivalent to almost 22 conventional aspirin tablets giving rise to a potentially acute toxic dose of salicylate [51]. Fortunately, the incidence of referrals due to MS has been in decline in recent years (Figure 1) with the overall fall being indicative of increasing numbers of alternatives, such as diclofenac and ibuprofen-based preparations. It is important to note that case reports involving MS, as a percentage of all issues involving topical agents, has stayed relatively constant at 10–11% over the past decade [13,14,15,16,17,18]. 

It must be acknowledged that the OTC status of many products containing MS can lead to the erroneous assumption that they are inherently safe, thereby leading to unintentional poisoning [56,57]. The latter is highlighted by the recent death of a seventeen-year-old cross-country runner after excessive self-administration of a topical muscle-pain relief treatment incorporating MS [58]. It has been estimated that over 25% of US parents have poor health literacy skills and there is little doubt that this will be a factor in some of these incidents—especially where administration and dosing will largely be done by the parent without medical consultation [57]. The American Association of Poison Control Centres Toxic Exposure Surveillance System (AAPCC-TESS) has reported that about 77% of the enquiries relating to exposures/incidents of MS poisoning involve children under six years of age [13,14,15,16,17,18]. 

Pharmaceutical preparations are not, however, the only source and there is increasing apprehension over the use of “natural” remedies involving various leaf and bark infusions [57,59,60]. Birch or wintergreen extracts, especially when distilled into the oil, were historically the principal source of MS and are little different from the synthetic “wintergreen” products available in any pharmacy. Infusions into hot water will yield the characteristic wintergreen aroma [22]. The sweet, distinctive aroma can too easily be associated with its use as a flavouring agent in confectionary [32], thereby reinforcing the perception that the substance is inherently safe to use. Moreover, the “age old” connotations ascribed to many of the formulations are often viewed as a safer, holistic alternative to modern drugs with consumer awareness benefiting from the proliferation of internet testimonials of the potential therapeutic benefit. Herbal supplements are an increasingly important contributor to OTC sales in western countries with year-on-year growth (6.8% in the US from 2013 to 2014) and it has been suggested that they are particularly significant among paediatric and adolescent populations [6,59,60]. Recent surveys revealed that between 0 and 17 years of age, the administration of herbal remedies in Germany and the US were relatively similar with 6% and 4%, respectively [59,60]. This increased public interest in traditional and herbal remedies now poses a significant concern as they invariably lack the conspicuous warnings and directions mandated for conventional product labelling [56,57,58]. While the need to provide clear, consistent, and standardized label information to support consumer comprehension has long been recognised for pharmaceutical products, the potency of natural components can be highly variable and efficacy will be highly dependent on the morphology, age, and quantity of the ingredients used. There can be a dearth of information on the safety of local (or internet) preparations where recipes are invariably vague. Casual inspection of web sites demonstrates an uncomfortably relaxed approach to hazard labelling. In many cases, the information presented invariably extols the virtues of the natural remedy without providing adequate caution as to the potential adverse health implications.

## 6. Technological Solutions

A prime issue in the adoption of topical MS products relates to the user administration of liquid formulations. While the instructions will invariably caution against over-application to the skin or direct ingestion, there is no physical barrier preventing either occurring should the user wish. Incorporation of MS in a patch or dressing is, however, an approach that mitigates against wilful misuse or inappropriate overuse and can effectively eliminate the possibility of accidental ingestion by children—historically a critical failing of wintergreen medicants [51,52,53,54,55,56,57]. There are a number of commercial forms that effectively lock the MS and co-drugs (such as menthol) within a framework that only permits transfer through transdermal contact. Passive diffusion of the therapeutic agents across the skin barrier can typically occur over a period of hours and they are invariably marketed as providing “long-lasting” pain relief. Such systems can vary in complexity from the agent being dissolved in adhesive binders that can be directly applied to the skin through to multilayer assemblies encapsulating a range of chemical components [61]. The latter can have profound impact on the performance of the patch and can include components that affect the transfer of the drug (permeation enhancers, rate controlling membranes, solubilizers), as well as enabling the design conformity of the device (adhesives, tackifiers, plasticisers) [61]. 

There remains considerable research into the design of supramolecular gelation layers than can encapsulate salicylates—and MS, in particular, with the aim of enhancing the biocompatibility of the host matrix and enabling more controllable release of the therapeutic agent [62,63,64]. While there are numerous formulations available, it is important to note that very few have been approved by the FDA for the temporary relief of pain relative to the many conditions associated with chronic pain, as indicated in Table 2.

The transdermal patches are generally considered to be first generation “devices” and almost invariably rely on the lipophilic properties of the drug to pass across the stratum corneum [65,66,67,68]. Although this action requires no additional input from the patient, it also means that there is no mechanism for modifying the rate at which the dose is delivered once the patch has been positioned. The main benefit of the patch system, however, is that it is effectively a “metered” dose, unlike the ointments, gels or liquids whose volume (and hence dose) depends principally on the judgement of the user. 

Although the drug delivery profile can, in principle, be controlled through the design of the patch structure, the rate of release can be further influenced by changes in ambient or local heat [66,68]. Elevated temperatures can accelerate the delivery of the drug but, in doing so, may also subsequently decrease the transfer rate once the heat source is removed—as a consequence of the load being prematurely depleted. The efficacy of the delivery can, therefore, be affected where such patches are intended to be applied over extended durations and where a suboptimal delivery arises in the later stages. Such profiles have been corroborated through a number of in vivo studies of patches containing methylphenidate [65], buprenorphine [66], and fentanyl [67,68]. The rate and extent of drug release, although dependent on drug formulation and patch design has, nevertheless, been shown to increase significantly upon the addition of heat. In the cases of buprenorphine and fentanyl, the addition of a heat pad led to plasma levels increasing by up to 55% and 61%, respectively [66,68]. 

### 6.1. Iontophoretic Delivery Options

**Figure 3:**
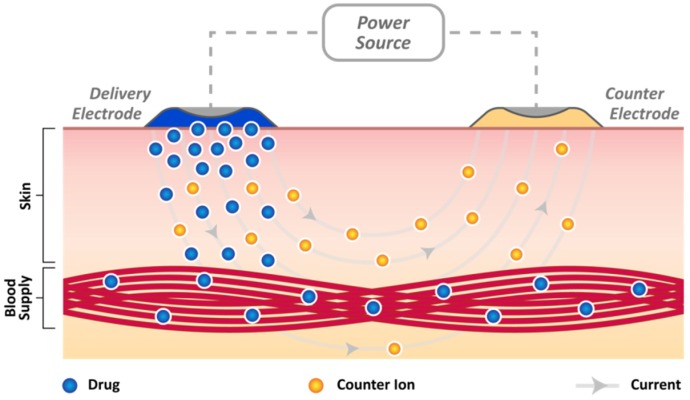
Electromigration during iontophoretic drug delivery.

The ability of small, neutral compounds to permeate the skin barrier has long been recognised and exploited, but the application of electrical current (iontophoresis), ultrasound (sonophoresis), or microneedle methods can provide options for enhancing the transfer [69,70,71,72,73,74,75,76,77,78,79]. Iontophoresis essentially employs a DC current to drive drugs across the stratum corneum, rather than provoke any significant disruption of the skin’s structure as indicated in Figure 3. These approaches are well established and there is an extensive literature available on their application to a wide spectrum of drugs and salicylates. A two-electrode system is almost invariably employed and placed directly on the skin surface as indicated in Figure 3. The “delivery” reservoir typically contains drugs bearing a charge similar to the polarity imposed at that electrode. The counter electrode completes the circuit and when the current is applied, ions migrate (driven) from the delivery side through the skin bridge, due to the electric field, with counter ions (ions of opposite polarity such as Na^+^ or Cl^−^) moving to restore charge balance. The efficiency of iontophoretic transport (ratio of current carried by the drug and the total current applied across the membrane) is usually low because of competition from a large pool of ions already present within the tissue and the relatively low electromobility of the target drug compared to the latter.

It has been shown that, with negatively-charged species, less than 20% of the current is carried by the compound [69,70,71]. Nevertheless, numerous studies have demonstrated the effectiveness of iontophoresis for enhancing the transport of salicylate [72,73], and there has been extensive research into minimising the effect of endogenous ion competition. The introduction of ion exchange membranes and drug carriers such as liposomes [74,75] microemulsions [76], polymeric nanoparticles [77], and solid liquid nanoparticles have all been investigated in order to enhance the drug transfer. The use of iontophoresis with lipid nanoparticles loaded with salicylic acid was shown to significantly improve the amounts delivered across human epidermal membranes in comparison to passive transfer—even when the latter was continued for four times the duration. Crucially, it has been shown that when applied to Wistar rats, the excised skin revealed that the salicylate concentration was greater in the skin and subcutaneous tissues directly below the iontophoresis delivery site than in the plasma, suggesting that the mode of action is local [78,79].

While there has been extensive investigation of iontophoretic salicylic acid transfer, methyl salicylate has received considerably less attention. The lipophilic properties of the ester and its ready absorption through the skin, in comparison to salicylic acid, has meant that there has been little need to drive its transfer. Nevertheless, Wani and Gaikwad (2013) investigated the effectiveness of employing iontophoresis in the delivery of MS to patients suffering with knee osteoarthritis (KOA) [80]. The patients were assessed for pain and functional capacity using the numerical pain rating scale (NPRS), walking speed test (WST), modified get up and go test (MGUGT), total single limb standing test (TSLST), and Western Ontario McMaster Universities Osteoarthritis Index (WOMAC) before and after two weeks of intervention. In comparison to a control group (receiving only a moist pack), significant improvements in all, except the MGUGT, scores were observed, suggesting that the approach was effective in enhancing pain relief and functional capability [80]. 

Leaving aside the therapeutic efficacy, the key benefit of iontophoresis on MS administration relates to the fact that it provides a mechanism through which the delivery rate may be controlled to a high degree of specificity. Due to the direct relationship between the applied current and transdermal flux, iontophoresis embodies the intrinsic ability to regulate or inhibit the delivery rate over a given period of time. Rate control may be devolved to the responsibility of the patient or, more ideally, controlled by incorporation of a microprocessor, thereby enabling complex and bespoke delivery profiles to be enacted. Given the increasing miniaturisation of electronic systems and integration with smart devices (phones, watches, and fitness trackers), it could then be expected that the translation of iontophoretic systems to a more manageable format is likely in the future [81]. The glucose watch, employing reverse iontophoresis has, in many respects, highlighted how the technology could be developed with the patient in mind and the need to accommodate everyday activities. While such devices can never compete with the low-cost disposable patches targeted at acute pain, the increased control over delivery schedule and potential optimisation of dose could be of significant benefit to those suffering from chronic conditions. Critically, such technology could ultimately provide the capability to titrate doses and prevent undesirable fluctuations of drug concentration in the blood, thus avoiding over-use.

### 6.2. Microneedle Systems

Microneedle (MN) patch systems have gained considerable interest and the scientific literature is awash with a multitude of designs covering an equally diverse range of drugs [82,83,84,85]. The key advantage of MN patches, from a drug delivery perspective, relates to their ability to painlessly breach the skin barrier [82]. As such, they are an ideal conduit for the transfer of agents which would otherwise have limited ability to absorb passively. There are numerous MN formats and a detailed description of their properties is beyond the scope of the present discussion. Irrespective of design, drug loading will be significantly limited in comparison to conventional oral (or intravenous) dosing and, therefore, they are almost invariably targeted at the delivery of low yield—high potency agents such as vaccines. There are extensive examples of their use in the delivery of diclofenac [82] but, as yet, no reports of salicylate transfer. The use of diclofenac is noteworthy beyond its use as an NSAID in that it has been shown to delay the closure of the transdermal channels created by MN patches [82,83]. This can be useful in “poke and patch” scenarios where topical agents applied as a gel or spray after removal of the MN exploit those channels to reach the dermis [77,78].

Microneedles that dissolve or swell are among the more recent developments within this field and can offer a means through which to control the rate of delivery. The drug is normally encapsulated as a composite component along with a structural material necessary to provide the needle framework. Upon insertion into the skin, the MN framework dissolves at a given rate (determined by the manipulation of formulation factors during manufacture) and, in doing so, releases the drug [77]. It could be envisaged that this strategy could be adopted for the delivery of salicylates and thereby offer a metered dose directly to the affected tissue. In the case of methyl salicylate, it does not offer much of an advance over the conventional passive patches and can still be prone to misuse where the patient is able to apply multiple patches. 

Microneedle technology has been evolving and, just as the passive patches are beginning to morph into smart systems capable of regulating dose, there is a slow transition from systems designed purely to ease drug delivery to integrated devices capable of sensor-actuator functions [82]. At present, the two functions are largely discrete, but it is inevitable that there will be an eventual marriage of the technology.

## 7. Conclusions

Transdermal drug delivery can often be regarded as a superior option to other routes of administration as it offers the prospect of greater control over the dosage. However, this comes with a multitude of caveats and there is no doubt that in acquiring greater control, the degree of complexity in the formulation or technological framework is dramatically increased. The development of smart technologies has gathered considerable pace, and while commercial devices have yet to gain a foothold, the foundations for their arrival have been laid. The convenience of the OTC passive patches and the fact that they can be worn, usually in an unobtrusive manner, and will deliver known amounts of MS over the course of several hours, already provides an excellent solution over the manual application of a topical gel, spray, or liquid. Upgrading to a wireless system controlled by a phone app brings further benefits, but it also significantly increases the cost. There may be limited efficacy in the latter for acute pain but there could be significant benefits in treating chronic pain where local or systemic concentrations can be regulated with greater precision. Generic iontophoretic patches for drug delivery are already available and it is inevitable that the software required to enable tailor drug profiles directed by a smart phone will follow.

## Figures and Tables

**Table 1:** Prevalence of methyl salicylate in consumer products.

Cosmetic Category	FDA Products	Conc. of MS %
Dentifrices	38	0.03
Mouthwashes and breath fresheners	49	0.08–0.2
Other oral hygiene products	6	0.2
Bath soaps and detergents	385	0.0001
Bath oils, tablets, and salts	124	—
Body and hand preparations	796	0.05
Skin cleansing	653	—
Douches	5	—
Foot powders and sprays	35	0.02
Hair conditioners	636	—
Shampoos	860	—
Tonics, dressings, hair-grooming aids	549	—
Paste masks	255	0.6
Skin fresheners	184	0.1
Other skin care preparations	692	0.02
Suntan gels, creams, and lotions	136	0.2

**Table 2:** FDA approved transdermal patches for pain relief.

Year	Drug	Product	Application
1990	Fentanyl	Duragesic	Chronic pain
1995	Epinephrine; Lidocaine HCl	Iontocaine	Local dermal analgesia
2005	Lidocaine; Tetracaine	Synera	Local dermal analgesia
2007	Diclofenac Epolamine	Flector	Nonsteroidal anti-inflammatory
2008	Menthol; Methyl Salicylate	Salonpas	Topical analgesic
2010	Buprenorphine	Butrans	Chronic pain
2013	Sumatriptan Succinate	Zecuity	Acute migraine pain
